# Using wireless technology in clinical practice: does feedback of daily walking activity improve walking outcomes of individuals receiving rehabilitation post-stroke? Study protocol for a randomized controlled trial

**DOI:** 10.1186/1471-2377-13-93

**Published:** 2013-07-18

**Authors:** Avril Mansfield, Jennifer S Wong, Mark Bayley, Lou Biasin, Dina Brooks, Karen Brunton, Jo-Anne Howe, Elizabeth L Inness, Simon Jones, Jackie Lymburner, Ramona Mileris, William E McIlroy

**Affiliations:** 1 Balance Mobility and Falls Clinic and Mobility Research Team, Toronto Rehabilitation Institute, University Health Network, Toronto, ON, Canada; 2 Heart and Stroke Foundation Centre for Stroke Recovery, Toronto Rehabilitation Institute and Sunnybrook Health Sciences Centre sites, Toronto, ON, Canada; 3 Department of Physical Therapy, University of Toronto, Toronto, ON, Canada; 4 Graduate Department of Rehabilitation Science, University of Toronto, Toronto, ON, Canada; 5 Department of Kinesiology, University of Waterloo, Waterloo, ON, Canada

**Keywords:** Stroke, Rehabilitation, Walking, Physical activity, Goal setting, Technology

## Abstract

**Background:**

Regaining independent ambulation is the top priority for individuals recovering from stroke. Thus, physical rehabilitation post-stroke should focus on improving walking function and endurance. However, the amount of walking completed by individuals with stroke attending rehabilitation is far below that required for independent community ambulation. There has been increased interest in accelerometer-based monitoring of walking post-stroke. Walking monitoring could be integrated within the goal-setting process for those with ambulation goals in rehabilitation. The feedback from these devices can be downloaded to a computer to produce reports. The purpose of this study is to determine the effect of accelerometer-based feedback of daily walking activity during rehabilitation on the frequency and duration of walking post-stroke.

**Methods:**

Participants will be randomly assigned to one of two groups: feedback or no feedback. Participants will wear accelerometers daily during in- and out-patient rehabilitation and, for participants in the feedback group, the participants’ treating physiotherapist will receive regular reports of walking activity. The primary outcome measures are the amount of daily walking completed, as measured using the accelerometers, and spatio-temporal characteristics of walking (e.g. walking speed). We will also examine goal attainment, satisfaction with progress towards goals, stroke self-efficacy, and community-integration.

**Discussion:**

Increased walking activity during rehabilitation is expected to improve walking function and community re-integration following discharge. In addition, a focus on altering walking behaviour within the rehabilitation setting may lead to altered behaviour and increased activity patterns after discharge.

**Trial registration:**

ClinicalTrials.gov NCT01521234

## Background

Stroke results in sensorimotor impairments, reduced balance control, and reduced aerobic function, which can limit the capacity for independent community ambulation [1-3]. Regaining independent ambulation is important to those with stroke [4,5] and is the most frequently-reported rehabilitation goal [6,7]. Independence in walking is important for maintaining autonomy and quality of life [5,8-10]. Improved functional outcomes are observed with intense [11-13], task-specific [14-18] rehabilitation that is delivered relatively early post-stroke onset [11,16]. Therefore, if a stroke patient wishes to improve walking ability s/he should do extensive walking practice, particularly early in his/her rehabilitation. In-patient rehabilitation provides a prime opportunity for individuals with stroke to practice walking such that they can ambulate safely post-discharge. However, the amount of daily walking reportedly completed by individuals with stroke during in-patient rehabilitation is low [19-22]. Importantly, the majority of walking bouts are of short duration (<1 minute) [19,20,22,23] and typically involve walking to essential activities (e.g. washroom, dining area, or therapy) [19].

**Figure 1:**
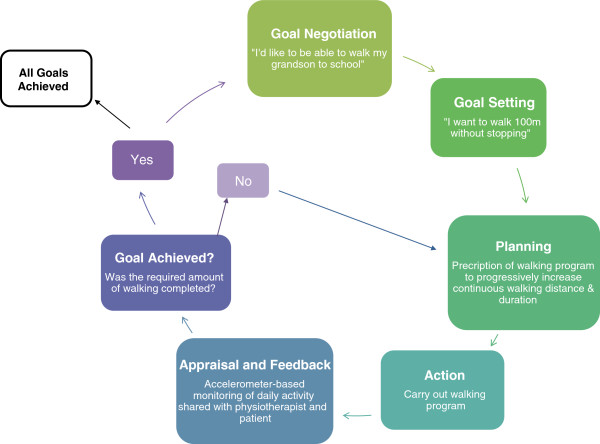
**Model showing the goal-setting and goal-planning process (modified from Scobbie et al., 2010; [**[27]**].** The patient may identify a broad walking goal upon admission to rehabilitation, such as “I want to be able to walk better”. The physiotherapist would discuss this goal with the patient and determine a more measurable and meaningful goal, such as “I want to be able to walk my grandson to school, which is 300m away from my house”. A short-term sub-goal is then established to help the patient progressively achieve their longer-term goal; i.e. “I want to walk 100m without stopping”. Following identification of short-term goals, a rehabilitation plan is developed and put into action. Progress towards the goal is appraised and feedback is provided to the patient; it is at this point in the goal-setting process that accelerometers can be beneficial. The appraisal will allow therapists and patients to determine if the sub-goal has been achieved. If so, a more challenging sub-goal may be developed. If the sub-goal was not achieved, the physiotherapist may modify his/her treatment plan. This cycle continues until all goals are achieved.

Goal setting is an essential part of rehabilitation but its use may be more ‘common sense’ rather than being grounded in sound psychological theory [24-26]. Scobbie and colleagues established recommendations for the implementation of goal-setting in rehabilitation based on three psychological theories (Figure 1): Goal Setting Theory, Health Action Process Approach, and Social Cognition Theory [25,27]. Within these recommendations, appraisal and feedback of performance were identified as important constructs [27,28]. Providing advice on health behaviours without objective feedback of compliance or outcomes may lead the patient to develop “cognitive fantasies”; i.e. belief that they are adhering to the advice when they are not [29,30]. Feedback of progress towards goals can increase motivation, improve self-efficacy and aid action-planning [28].

Researchers have begun to investigate the utility of ambulatory monitoring using accelerometers [31]. We developed the Accelerometry for Bilateral Lower Extremities (ABLE) system to specifically monitor daily walking activity in individuals with stroke [19,22]. The ABLE system combines inexpensive ‘off the shelf’ accelerometers with custom written software and provides information of each individual bout of walking completed throughout the patients’ day. Therefore, the system can provide feedback to the patient or therapist on not only the total amount of walking completed in the entire day, but also the duration of each walking bout (i.e. how long the patient walked continuously) and times of day when the patient was active or inactive. Furthermore, by placing accelerometers on both lower limbs, we can provide information regarding the control of walking throughout the day (i.e. temporal features of gait). This information can be provided to the patient and his/her physiotherapist as part of a goal-setting process in order to determine whether the patient’s goals for ambulation are met.

The primary objective of this study is to determine if feedback about characteristics of daily walking activity, provided to patients and their treating therapists, will increase walking activity in a group of individuals receiving rehabilitation post-stroke. We hypothesize that, compared to a control group who do not receive feedback via the treating physiotherapists, those who receive accelerometer-based feedback of daily walking activity as part of a goal-setting process will show: increased total daily walking activity, as measured by number of steps per day, total duration of walking activity, and total distance walked; increased frequency of ‘long’ walking bouts (i.e. > 5 minutes in duration); and improved control of walking, as defined by increased self-selected walking speed, and improved symmetry in spatio-temporal characteristics of walking. While walking practice should be initiated during in-patient rehabilitation, problems with walking function may not be fully apparent until the patient is discharged home. Therefore, we will examine the effects of accelerometer-based feedback administered during both in- and out-patient rehabilitation. Under the assumption that accelerometer-based feedback will increase walking activity, the secondary objective of this study is to determine if increased walking activity during rehabilitation results in increased rates of goal attainment, increased satisfaction with rehabilitation, improved self-efficacy, and improved community re-integration post-discharge from rehabilitation.

## Methods

### Design overview

**Figure 2:**
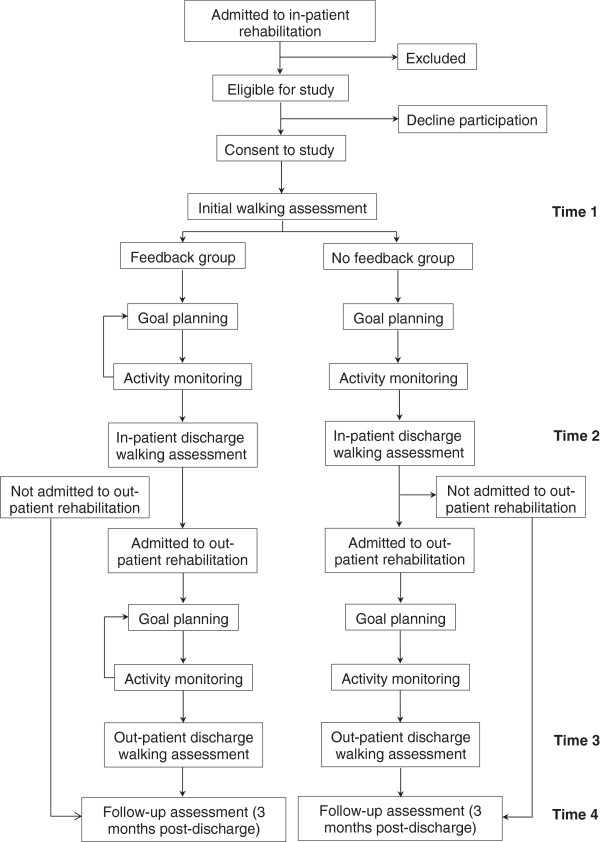
**Participant flowchart.** The study will span three phases of rehabilitation and recovery post-stroke: in-patient rehabilitation, out-patient rehabilitation, and post-rehabilitation community re-integration. Patients will be assessed for eligibility upon admission to in-patient rehabilitation and throughout their stay. Upon providing consent, participants will be assigned to either the feedback or no feedback group. Participants who are discharged to out-patient rehabilitation at one of Toronto Rehab sites will continue to complete the schedule of goal-planning for walking activity either with or without feedback (depending upon group allocation).

This is a single-blind randomized controlled trial with the assessor being blinded to the group allocation (participants cannot be blinded to group allocation). Participants will be randomly assigned, using allocation concealment, to one of two groups: feedback or no feedback. The study will span three phases of rehabilitation and recovery post-stroke: in-patient rehabilitation, out-patient rehabilitation, and post-rehabilitation community reintegration. Outcome measures will be obtained at up to four time points (Figure 2): 1) upon entry into the study; 2) immediately prior to discharge from in-patient rehabilitation; 3) immediately prior to discharge from out-patient rehabilitation (for those admitted to out-patient rehabilitation at the institution); and 4) three months following discharge from rehabilitation. This study was approved by the University Health Network research ethics board (protocol number 11-027-DE).

### Participants

Individuals with sub-acute stroke who are attending in-patient rehabilitation at Toronto Rehab will be recruited to this study and will provide written informed consent prior to participating. All participants will have identified improving walking function as a rehabilitation goal upon admission and will be able to walk without supervision at the time of recruitment into the study. Individuals unable to provide consent to participate in the study due to foreign language or cognitive impairment will be excluded. In order to ensure generalizability to the wider stroke population, no further exclusion criteria will be applied.

### Group allocation and blinding

Participants will be assigned using a blocked stratified randomization approach to one of two groups: 1) feedback of walking activity; or 2) no feedback of walking activity (i.e. control group). There will be two strata based on the factor walking speed. Walking speed predicts the level of community ambulation achieved following discharge from rehabilitation [5]. A threshold walking speed of 0.42 m/s has been found to be predictive of community ambulation post-stroke [32]; therefore, participants will be stratified according to self-selected walking speed (slow walkers: <0.42 m/s; moderate-fast walkers: ≥0.42 m/s). Stratification will help to ensure that the two groups do not differ significantly on this factor [33,34] and that there will be approximately equal numbers of participants assigned to each group. The block size within strata will be four participants.

Group allocation will be performed by the principal investigator. A computer-generated random sequence will be used to assign participants. The treating physiotherapists will administer the goal-setting/planning (including feedback, if appropriate). A blinded research assistant will conduct the assessments and process data. Participants cannot be blinded to group allocation and will be informed of the chance of being allocated to one of two groups.

### Current practice for goal setting and goal planning at Toronto Rehab

Goal setting and planning form an important part of care at Toronto Rehab. Goal setting is a patient-centred and collaborative process [27,28,35,36] wherein, upon admission to rehabilitation, the patient discusses goals with the interprofessional care team. Goals identified at this stage of care might be broad; e.g. “I want to be able to walk better”. Through discussion, the team explores the relationship of the individual’s goal and the impact of their condition within the context of participation and important life roles; e.g. “I want to be able to continue to provide childcare to my grandson, including walking him to school.” This part of the goal-setting and planning process is often termed ‘goal negotiation’ [27]. The individual therapists then work with the patient to identify specific and challenging [24,28] sub-goals at the activity or impairment level that would lead to progressive achievement of the larger goal; e.g. “I want to be able to walk 100m without stopping”. Each individual therapist then bases their therapeutic intervention on the patient’s identified goals. The goal coordinator, who is a member of the interprofessional team, meets with the patient once every two weeks to discuss progress towards all of his/her goals. Each individual therapist also meets with the patient routinely during daily therapy sessions to discuss progress relevant to his/her practice; i.e. physiotherapists would discuss progress towards walking goals with patients on a regular basis. During either the fortnightly meetings with the goal coordinator, or discussions with the patient’s therapists, sub-goals may be re-evaluated depending upon how the patient is progressing towards his/her goals; e.g. a more challenging goal may be set if previous sub-goals have been achieved. Specific goals are documented for each patient in the clinical chart. Throughout rehabilitation and at the time of discharge, the individual therapist or goal coordinator determines if the patient’s goals were achieved (see Figure 1).

### The ABLE system – description, on-going development and pilot implementation

**Figure 3:**
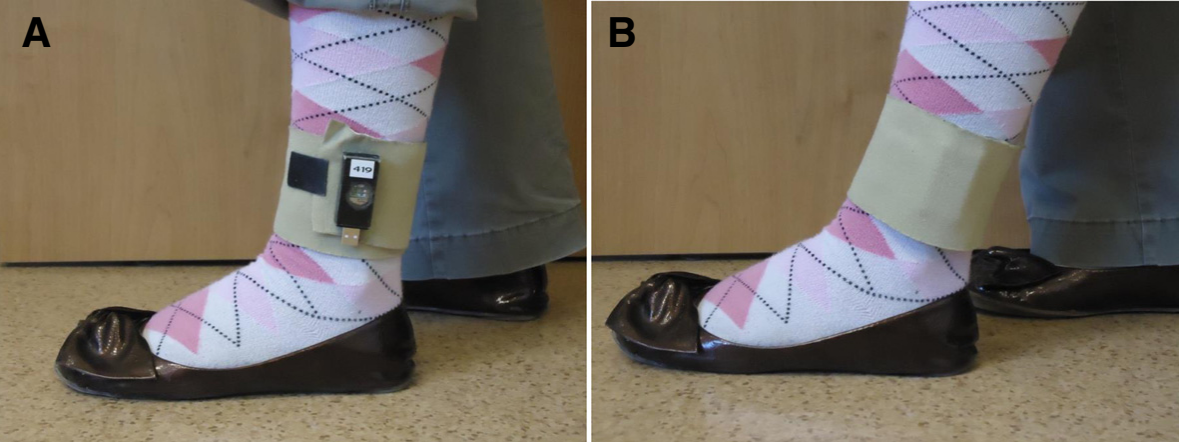
**The ABLE accelerometers.** The ABLE accelerometer as affixed to the ankle above the lateral malleolus. Model X6-2mini is shown. In panel **A**, the accelerometer is exposed to show its size. In panel **B**, the accelerometer is covered by the foam wrap; this is how the accelerometers are affixed in practice.

The ABLE system consists of two lightweight commercially-available triaxial accelerometers (Figure 3; Model X16-1C or X6-2mini, Gulf Data Concepts, LLC., Waveland, Mississippi, USA). The accelerometers are ‘self-recording’, such that data are stored directly on an internal microSD card without the need for an external data logger [19,37]. The accelerometers are affixed just above the ankles of both limbs using a breathable foam wrap that is Velcro® hook receptive (Pro Wrap, Fabrifoam, Exton, Pennsylvania, USA). The accelerometers record acceleration in three axes for the entire monitoring period in blocks of eight hours at 40 Hz. Our previous work [19] confirms that these parameters are sufficient for the purpose of whole-day ambulatory monitoring. Participants are instructed to only remove the accelerometers before going to sleep at night (to prevent discomfort), when showering, and when required to do so for medical testing (e.g. magnetic resonance imaging).

Data are transferred from the accelerometers and processed using a custom-written algorithm [19] (MatLab, The Mathworks, Natick, Massachusetts, USA). The raw acceleration data are high-pass filtered at 0.25 Hz to remove the gravity component [38] and low-pass filtered at either 5 Hz for the purpose of step detection or 10 Hz to detect the temporal features of each step (i.e. heel strike and toe off) [19]. A bout of walking is defined by at least 4 consecutive steps with <10 s between steps; if there is a pause of 10 s or more between steps a new bout is identified. The following measures are calculated for each walking bout: number of steps taken, total bout duration, average cadence (steps per minute), and average swing duration (heel strike minus toe off) for each limb. Heel-strike and toe-off detection has previously been validated against foot-switches [19]. On-going development aims to determine the validity of calculating step length, walking speed and distance walked for each bout.

### Intervention

Participants will be recruited into the study when they are deemed to be ambulatory without supervision. Patients with stroke tend to be discharged from in-patient rehabilitation 2–4 weeks after they reach this status. Therefore, the length of the in-patient intervention phase will vary between participants but will be approximately 2–4 weeks long. Out-patient rehabilitation services are offered in three blocks of 4–6 weeks duration; the first block is 6-weeks long to allow extra time for assessment prior to prescription of rehabilitation services. Participants in this study will be followed only for the first and second (if applicable) block of out-patient rehabilitation. The third block is often either not completed at all or not completed immediately subsequent to the second block (i.e. there may be a gap of a number of months between the second and third block). Therefore, the duration of the out-patient phase of the intervention is 6–10 weeks.

In order to ensure that all physiotherapists administer the intervention in a consistent manner, we will host a series of in-services concerning goal-planning of walking activity. These in-services will focus specifically on the output from the ABLE system, which is the new component of this study. Physiotherapists will learn how to interpret the output from the ABLE system and how to use this information to evaluate a patient’s progress towards their goals. Specific cases will be discussed based on pilot testing of ambulatory monitoring.

#### In-patient rehabilitation phase

Participants in both intervention groups will meet with their primary treating physiotherapist once per week to discuss progress towards their goals for rehabilitation that are relevant to physiotherapy, including goals for walking. All participants will wear the accelerometers every weekday during in-patient rehabilitation to monitor daily walking activity. Research personnel will meet with the patients each morning to ensure that the accelerometers are worn correctly.

For those participants assigned to the feedback group, physiotherapists will receive a daily summary of patients’ walking activity as a tool to guide goal planning. Physiotherapists will use the information as a ‘homework checker’ to determine if patients are complying with an assigned walking program. In the case of non-compliance, the physiotherapist will discuss a coping strategy for better integrating walking activity into the patients’ day. In the event that the patient is meeting their specific sub-goals for walking activity, the physiotherapist will re-evaluate these sub-goals and suggest more challenging goals.

For participants assigned to the control group, physiotherapists will not receive accelerometer-based feedback of daily walking activity. However, physiotherapists will still discuss the achievement of walking goals with their patient. The same strategies implemented in the feedback group will be implemented in the control group (i.e. action plans, coping strategies, re-evaluation of goals). This process is no different to usual care around goal planning at Toronto Rehab.

#### Out-patient rehabilitation phase

Participants in both groups will meet with their primary treating physiotherapist regularly to discuss progress towards walking goals. All participants will wear the accelerometers daily during out-patient rehabilitation. For those in the feedback group, physiotherapists will receive a weekly summary of patients’ walking activity as a tool to guide goal planning. Participants in the control group will attend goal-planning meetings with their physiotherapists, as is usual care.

### Measures

A summary of the measures collected at each point in the study is included in Table 1.

**Table 1:** Measures collected at the assessment time points

	**Time 1: study enrolment**	**Time 2: discharge from in-patient rehabilitation**	**Time 3: discharge from out-patient rehabilitation**	**Time 4: three months post-discharge from out-patient**
Demographic information	✓			
Stroke information	✓			
Medical conditions	✓	✓	✓	✓
Falls	✓	✓	✓	✓
Chedoke-McMaster Stroke Assessment	✓	✓	✓	
Berg balance scale	✓	✓	✓	
Activity-specific balance confidence questionnaire	✓	✓	✓	
National Institutes of Health Stroke Scale	✓			
Ambulatory monitoring	✓ (3 days)	✓ (3 days)	✓ (7 days)	
Spatio-temporal features of walking	✓	✓	✓	
Stroke self-efficacy questionnaire	✓	✓	✓	✓
Goal attainment		✓	✓	
Satisfaction with progress towards goals		✓	✓	
Community integration questionnaire			✓	✓
Barriers to walking interview	✓	✓	✓	✓

#### Cohort descriptors – demographics, medical & stroke history, and measures of physical function

The following demographic and medical information will be obtained from clinical charts on admission to rehabilitation in order to characterize the study cohort: age, sex, height, weight, date of stroke onset, lesion location, pre-morbid medical history & current medical conditions, prescription medications, the National Institutes of Health Stroke Scale (NIH-SS) [39], the Chedoke-McMaster Stroke Assessment (CMSA) [40] foot and leg scores, Berg Balance Scale (BBS) [41], and the Activity-specific Balance Confidence (ABC) questionnaire [42]. The NIH-SS is an 11-item scale that provides a gross measure of the effects and severity of stroke. The NIH-SS has shown good intra-rater (intraclass correlation coefficients; ICCs = 0.93) and inter-rater (ICCs = 0.95) reliability [43]. The CMSA assigns a score according to the level of motor recovery in the foot and leg and is frequently used to evaluate level of motor recovery post-stroke in clinical settings. The CMSA foot and leg scores have good intra-rater (ICCs = 0.94-0.98) and inter-rater (ICCs = 0.85-0.96) reliability [40]. The BBS is a 14-item observational rating scale that provides a measure of functional balance. Participants are asked to perform each of the 14 tasks and their ability to perform the task is rated on a scale from 0–4. The BBS shows good internal consistency (Cronbach’s α=0.92-0.98) and good inter-rater (ICCs = 0.95-0.98), intra-rater (ICCs = 0.97) and test-retest (ICC = 0.98) reliability post-stroke [44]. The ABC is a 16-item questionnaire that asks individuals to rate confidence performing various daily activities. The ABC shows good internal consistency (Cronbach’s α=0.94) and test-retest reliability (ICC = 0.85) in individuals post-stroke [45].

In addition to collection at admission to rehabilitation, medical conditions will be reviewed at all assessment time points to confirm that there have been no major changes in medical status between assessments. The CMSA, BBS, and ABC will be repeated at discharge from in-patient and out-patient rehabilitation as impaired balance control, impaired lower-limb function and reduced balance confidence could potentially be barriers to independent ambulation. The occurrence of falls will be documented for safety monitoring purposes.

#### Primary outcomes – daily walking activity & control of walking

The primary outcome measures pertain to walking activity as averaged over three days around the first two assessment time points (i.e. enrolment into the study and discharge from in-patient rehabilitation), and over seven days before the last assessment time point (discharge from out-patient rehabilitation). A longer monitoring period will be used for the out-patient phase as patients have more options for mobility post-discharge. Summary, whole day, outcome measures will be used in the analysis; i.e. total walking duration, total number of steps taken and total distance walked. However, it is most relevant to community ambulation and aerobic fitness to show increased duration of continuous walking [46,47]; therefore, we will also calculate the frequency and duration of longer walking bouts (i.e. >5 minutes continuously). Furthermore, intensity of walking is also important for cardiovascular benefit; therefore we will determine the frequency and duration of ‘high intensity’ walking, defined by either a walking speed or cadence ≥ 85% of maximum walking velocity.

Increased walking practice should improve control of walking. Spatio-temporal features of self-selected walking will be measured using a 4 m-long pressure sensitive mat (GAITRite, CIR Systems, Inc, Havertown, Pennsylvania, USA). Participants will start walking at least 1 m away from the mat and will be instructed to walk across the mat until they reach a mark 1 m from the end of the mat. Participants will be instructed to walk at their normal pace. Participants will complete three passes across the mat and measures will be averaged across the three passes. Walking speed, cadence, step length, and symmetry of spatio-temporal measures [48] will be calculated. While it is possible to determine these measures from the daily walking activity collected using the ABLE system, standardized assessment is required for pre- to post-intervention comparison of spatio-temporal measures.

#### Secondary outcomes – self- efficacy, community integration, goal attainment, satisfaction & barriers

The Stroke Self-Efficacy Questionnaire (SEQ) [49] will be used to measure stroke-specific self-efficacy. This 13-item questionnaire asks participants to rate confidence in completing various tasks, including walking indoors and outdoors and exercising, on a scale from 0–10. This questionnaire shows good test-retest reliability (ICCs > 0.87) [50], internal consistency, and criterion validity and can discriminate between patients who are independently ambulating and those who are not [49].

The Community Integration Questionnaire (CIQ) will be used as a measure of integration and participation post-discharge from in- and out-patient rehabilitation [51,52] and has been used often in stroke research [53-56]. This is a 15-item questionnaire that evaluates integration into the community in three domains: home, social, and productive activities (e.g. employment or volunteer activities). Researchers have developed a version of the CIQ specifically for individuals with aphasia and this version of the questionnaire shows excellent test-retest reliability in the post-stroke population (ICC = 0.96) [55].

Goal attainment will be determined from patient charts at discharge from both in-patient and out-patient rehabilitation. Goals are classified ‘achieved’, ‘partially achieved’, ‘not completed’, or ‘discontinued’. Satisfaction with progress towards goals will also be extracted from patient charts (rated on a 10-point scale). We will interview patients in order to determine barriers to walking. A single open-ended question will be used: “what, if anything, do you think is preventing you from walking more than you are at the moment?”.

### Statistical analyses and sample size

The following potentially confounding measures will be compared between the two intervention groups: CMSA, BBS, NIH-SS, ABC, and length of stay (in-patient rehabilitation). Measures that differ significantly between the two groups will be included as covariates in statistical models comparing interventions. Repeated-measures analysis of variance (ANOVA) or analysis of covariance (ANCOVA; as appropriate), with group-by-time interaction, will be used to evaluate the effect of the interventions on walking activity, spatio-temporal characteristics of walking, and SEQ. Separate ANOVAs will be used for each outcome measure. The ‘group’ term is the intervention group; the ‘time’ term is the assessment time point. The group-by-time interaction effect will reveal if there is a greater change over time in one group compared with the other. Separate analyses will be conducted to compare measures at the first three time points; i.e. discharge from in-patient will be compared to study enrolment, and discharge from out-patient will be compared to discharge from in-patient. One-way ANOVA or ANCOVA will be used to compare the CIQ and satisfaction between the two groups at discharge from rehabilitation and three months post-discharge from in-patient. The frequency of goal attainment between the two groups will be compared at discharge from both in-patient and out-patient rehabilitation; intent-to-treat analysis will be employed for this outcome for all participants initially randomized, including those who withdraw from the study [24,57]. Barriers to walking will be documented.

For sample size estimates, we used change in total walking duration from the initial assessment to discharge from in-patient rehabilitation (i.e. the first phase of the study) as our primary outcome of interest. In our pilot work, we observed an average increase in total daily walking activity of 20 minutes from the initial assessment to discharge in a group of stroke in-patients. We expect that an average increase in daily walking activity of 40 minutes in the feedback group would be clinically meaningful (i.e. 20 minutes more than expected with no feedback). The standard deviation for change in daily walking time was 23 minutes in our pilot work. Using a sample size formula for repeated measures ANOVA [58], an improvement of 20 minutes of walking more in the feedback group than the no feedback group, a standard deviation of 23 minutes, a probability of a Type I error of 0.05 and probability of Type II error of 0.1, 28 individuals per group will be required in the final analysis. In order to achieve a final sample of 56 (i.e. 28 per group) we will aim to recruit approximately 62 participants (i.e. assuming that approximately 10% of participants will withdraw from the study). Sample size and power estimates using an updated standard deviation will be reassessed after the first 12 participants have completed the first phase of the study, as per CONSORT recommendations [59,60].

## Discussion

While there has been interest in using accelerometers for walking-related goal-setting and goal-planning in rehabilitation, the effectiveness of this technology in clinical practice to improve walking activity has not been shown. This study may demonstrate that accelerometer-based feedback of walking activity can increase walking activity, improve walking outcomes, and potentially improve community re-integration following discharge from stroke-rehabilitation. The study design deliberately integrates the novel intervention into current practice; therefore, the results will be immediately generalizable to other stroke rehabilitation settings.

## Abbreviations

ABC: Activity-specific balance confidence questionnaire; ABLE: Accelerometry for Bilateral Lowe Extremities; ANCOVA: Analysis of co-variance; ANOVA: Analysis of variance; BBS: Berg balance scale; CIQ: Community integration questionnaire; CMSA: Chedoke-McMaster Stroke Assessment; ICC: Intraclass correlation coefficient; SEQ: Stroke self-efficacy questionnaire.

## Competing interests

The authors declare they have no competing interests.

## Authors’ contributions

AM conceived of the study and drafted the manuscript. All authors participated in the design of the study and helped to draft the manuscript. All authors read and approved the final manuscript.

## Pre-publication history

The pre-publication history for this paper can be accessed here:

http://www.biomedcentral.com/1471-2377/13/93/prepub
